# Adolescents’ reactions to participating in ethically sensitive research: a prospective self-report study

**DOI:** 10.1186/s13034-015-0074-3

**Published:** 2015-08-21

**Authors:** Penelope Hasking, Ruth C. Tatnell, Graham Martin

**Affiliations:** School of Psychology and Speech Pathology, Curtin University, GPO Box U1987, Perth, WA 6845 Australia; Department of Psychiatry, Monash University, Melbourne, Australia; Centre for Clinical Psychiatry and Neuroscience, The University of Queensland, Herston, Australia

**Keywords:** Ethics, Adolescents, Mental health

## Abstract

**Background:**

Conducting psychological research with adolescents is imperative for better understanding, prevention and treatment of mental illness. However there is concern that research addressing topics such as mental illness, substance use and suicidality has potential to distress participants, particularly youth.

**Method:**

We administered a questionnaire to 1973 adolescents (13–18 years) at two time points, one year apart. Participants responded to items regarding nonsuicidal self-injury, psychological distress, history of physical and/or sexual abuse, adverse life events, alcohol use, suicidal behaviour, self-efficacy, and coping skills as well as two open-ended questions regarding whether they enjoyed participating in the research and whether participation worried or upset them.

**Results:**

Most youth (74 %) enjoyed participation and cited altruistic reasons and a greater self-awareness as reasons. Those reporting being upset by the questionnaire (15 %) reported poorer psychological functioning than their peers. Youth who were upset by their participation at baseline, but who reported enjoying the questionnaire at follow-up reported improved psychosocial functioning over time, while the reverse was true for those who initially enjoyed participation but later reported the questionnaire upset them.

**Conclusions:**

Results suggest researchers acknowledge benefits for young people who participate in research, but also be mindful of the potential for distress among the most at risk youth.

## Background

Research regarding mental illness, substance use, nonsuicidal self-injury (NSSI) and suicidal behaviour is crucial to evidence-based prevention, early intervention and treatment efforts. Many signs of psychological distress or risk-taking behaviour (e.g. NSSI) first emerge in adolescence, and one in five young people will be diagnosed with a mental disorder before they reach adulthood [1]. As such, research which seeks to model risk and protective factors for later psychological distress among currently healthy adolescents is vital to better understanding the aetiology of mental disorders, and to development of effective prevention and early intervention strategies to interrupt negative psychological trajectories resulting from early distress.

However such research raises significant ethical challenges. Even among psychologically healthy participants, questions regarding NSSI, psychological distress, substance use, and suicidal behaviour may cause emotional distress. Developmental psychologists argue that adolescence is a particularly important time for the development of emotional maturity, emotion recognition and regulation, and adaptive coping skills [2]. Thus, while the majority of young people are resilient, adolescents may have a limited capacity, relative to adults, to reflect on sensitive topics without experiencing some degree of distress [3].

Despite concerns of research ethics committees and institutional review boards (IRBs), rather than experiencing distress, it is possible that young people value participating in research and that they derive benefit from doing so [4, 5]. We explored these issues as part of a larger study which aimed to identify risk and protective factors for the development of later psychological distress and NSSI among adolescents.

### Guidelines for the ethical conduct of research

In Australia, where the current study was completed, conduct of research is underpinned by the values set out in the National Statement on Ethical Conduct in Human Research [6], guided by the Declaration of Helsinki [7]. The National Statement articulates that research with young people “should provide for the child or young person’s safety, emotional and psychological security, and wellbeing” (p56). Inherent within this is that researchers should minimise any potential for distress to arise. The National Statement also distinguishes between harm, discomfort and inconvenience. Specific examples of psychological harm include: “feelings of worthlessness, distress, guilt, anger or fear…disclosure of sensitive or embarrassing information” (p16). While ethical review boards serve an important, and vital, role in protecting both participants and researchers, concern regarding the impact of asking young people mental health questions, with potential to cause distress, can result in research being hindered in obtaining approval from the appropriate governing bodies (i.e., IRBs) [8, 9].

### Effects of participating in research

A growing body of research has explored the issues of harm and psychological distress as a result of research participation, most notably in relation to trauma and suicidal behaviour. The consensus has been that participation is not distressing, or is distressing only for a minority [10]. Among a sample who had recently been physically or sexually abused, Johnson and Benight [11] noted only 6 % of their participants regretted participating and 45 % found involvement beneficial. However these studies have primarily focused on what might be considered ‘high-risk’ groups of participants, including victims of child abuse [12], participants reporting recent physical or sexual abuse [11], or prison inmates who had attempted suicide [13].

Less research has explored the impact of asking sensitive questions of healthy adolescent samples. Gould et al. [14] and Robinson et al. [5] both assessed responses to a screening program to detect students at risk of psychological problems and suicide, administered to whole classrooms of school-based adolescents. Screening was not associated with change in suicidal ideation [14], or reported distress [5], suggesting asking these questions does not increase suicidality, or cause notable changes in mood. In fact, Gould et al. [14] observed that students identified as ‘high risk’ (those with depressive symptoms, substance abuse and/or previous suicide attempt) reported *less* distress and suicidal ideation following the screening than ‘high risk’ students who did not receive screening.

### The current study

Our key aim was to establish whether adolescents who participate in research, designed to assess NSSI and related psychological constructs, find it enjoyable or upsetting, and reasons for this reaction. We also sought to establish whether reactions to participation were associated with psychosocial functioning among adolescents over time. To this end we assessed whether participants enjoyed participating in our study and whether anything worried or upset them, and explored how these responses were related to psychosocial functioning (e.g. self-esteem, coping skills, optimism, adverse life events, alcohol use, mental health, and NSSI). We then tested whether changes in these factors, over a 1-year period, were related to changes in whether participants reported enjoying the questionnaire or being upset by the study. To do this we examined changes in the psychosocial variables over time and how these differed for students who enjoyed participation at both time points, those who were worried or upset at both time points, and those who changed their responses over time (e.g. enjoyed the study initially but found it upsetting at time 2). Finally we assessed whether being upset by the questionnaire was associated with later onset of NSSI. We expected that participants who reported being worried or upset would report poorer psychosocial functioning. Further we expected deterioration in psychosocial functioning among students who initially enjoyed the questionnaire but later found it upsetting, and improvement in psychosocial functioning over time for those who were initially upset but later enjoyed participation.

## Method

### Participants

Participants were recruited from 40 secondary schools in five Australian states/territories to participate in a larger study on how adolescents cope with emotional problems. Parents of all students enrolled in grades 7–10 at participating schools were sent information about the study and invited to consent to their child’s participation (one information sheet addressed to parents/guardians was sent home with each child; n = 14,841). Consent forms were returned by 27.8 % (n = 4119) of parents and 75.6 % granted consent for study participation (n = 3116; 21.0 % total sample), a rate consistent with previous studies requiring active parental consent in Australia [15]. Of the students with parental consent, 2637 completed questionnaires at baseline.

Of these participants, 1973 completed the questionnaire one year later (*M* = 11.7 months, sd = 1.05), a retention rate (75 %) similar to other longitudinal studies with adolescents [16]. Only participants providing data at both time points were included in analyses. Reasons for attrition included transfer to another school (*n* = 96), school withdrawal (*n* = 114), parent/student withdrawal (*n* = 26), deceased (*n* = 1), or not present at second questionnaire administration (*n* = 428). All participants were between 12–18 years at baseline (*M* = 13.89, sd = 0.97).

## Materials

Two items assessed reactions to completing the questionnaire. These were: “Did you enjoy completing the questionnaire (yes/no); Why or why not?” and “Did anything in this questionnaire worry or upset you (yes/no)”. If they were worried or upset participants were asked “What was it that worried or upset you?” In addition, participants completed the following measures:

Part A of the Self-Harm Behavior Questionnaire (SHBQ) [17], was used to assess NSSI, and, if present, the method, recency, frequency and severity (from not at all serious to life-threatening) of the behaviour. Participants were also asked whether they had thoughts of taking their life, and if they had ever tried to take their own life. The SHBQ has acceptable internal consistency across young adults and adolescents in community samples (0.89–0.96) [17, 18] and differentiates between suicidal and non-suicidal young people [18]. Cronbach’s alpha in the current sample was 0.88.

The Adolescent Life Events Scale (ALES) [19] asks about individuals’ experience of each of 20 items (e.g. problems keeping up with school work, death of a family member), with the response options; never, yes more than a year ago, and yes within the past 12 months. The scale has good face validity, and in this sample a Cronbach’s alpha = 0.75.

The Alcohol Use Disorders Identification Test (AUDIT) [20] consists of three subscales assessing frequency and quantity of consumption, dependence, and alcohol-related problems respectively. The current research utilised only the consumption scale due to the participants’ age. Cronbach’s alpha in this sample was 0.91.

The General Health Questionnaire (GHQ-12) [21] is a 12 item measure to assess psychological distress, including anxiety and depression. Baksheev et al. [22] have demonstrated validity with a sample of Australian high school students. Cronbach’s alpha = 0.91 in the current sample.

The Rosenberg Self Esteem Scale (RSES) [23] comprises 10 items with equal numbers positively and negatively worded. This scale has good face validity, convergent and discriminant validity as well as internal consistency and reliability [23]. The Cronbach’s alpha in this sample was 0.89.

The General Self-Efficacy Scale **(**GSE) [24] was developed as a subjective measure to assess people’s perceptions of their own efficacy, in a broad, context free scenario. The GSE has been administered in many countries and shows high reliability, construct validity and stability over time and across different cultural groups. In this sample Cronbach’s alpha = 0.85.

The Life Orientation Test-Revised (LOT-R) [25] is a measure of participants’ optimism. The 10-item scale has acceptable stability and internal consistency. The scale also possesses good convergent and discriminant validity [26], and a Cronbach’s alpha of 0.71 in this sample.

The Adolescent Coping Scale (ACS) [27] short form consists of 18 items assessing three primary factors: problem solving, reference to others, and non-productive coping. The scale shows acceptable test–retest reliability, and predictive validity [27]. The Cronbach’s alpha’s for each of the subscales in the current sample were; problem solving 0.68, reference to others 0.72, and non-productive coping 0.36.

### Procedure

Ethical approval to conduct the project was obtained from Human Research Ethics Committees at Monash University and the University of Queensland, as well as all educational jurisdictions involved. Explanatory statements and consent forms were distributed to prospective participants by schools. Adolescents who obtained parental consent provided their own written consent to complete the 1-h questionnaire at school, during school hours. All participants were informed they could withdraw from the study at any time. All participants consented to participation and publication of the findings. Participants were supplied a unique code to ensure confidentiality, but enable identification if responses indicated immediate concern for life. Upon completion, all participants were given an information pack with mental health resources. This procedure was repeated at follow-up.

### Data analysis

Chi square tests confirmed that whether participants enjoyed the survey or were upset by it did not vary by geographic location, remoteness, SES of school or religiosity of students (all *p* > 0.05). Consequently these demographic variables were not controlled in subsequent analyses.

Two mixed model MANOVAs were performed to determine if changes in psychosocial functioning were associated with enjoying the questionnaire or being upset by the questionnaire. In the first MANOVA, participants were divided into four groups: (1) those who enjoyed the study at both times, (2) those who did not at either, (3) those who enjoyed it the first time, but not the second, and (4) those who did not enjoy the first administration, but did the second. The second MANOVA assessed the change in whether participants were worried or upset by the questionnaire (four groups: upset at both times, not upset at either time, those who upset at baseline but not follow-up, and those who were not upset at the first administration, but were at the second). Both MANOVAs assessed group differences at each time point (between group factor), changes over time on the psychological variables (within group factors), and any differential changes over time according to group membership (i.e. interaction between within- and between-group factors).

Chi Square statistics were used to explore the relationships between responses to the two key questions and specific life events. Finally, we explored whether enjoying the questionnaire or being worried about it was related to onset, maintenance or cessation of NSSI over the study period. Onset was recorded when participants reported no history of NSSI at baseline, but reported NSSI within the last 12 months at follow-up. Participants reporting no NSSI in the last 12 months at follow-up (but recorded a prior history) were considered to have ceased the behaviour. Participants reporting a history of NSSI at both times points (within last 12 months at follow-up) were considered to have maintained NSSI. To minimize the impact of Type I error alpha was set to 0.01 for all analyses.

Qualitative responses were coded using thematic analysis [28]. Initial codes were collated into themes, and refined through discussion among the authors. 20 % of responses were independently coded by a researcher unaware of the study aims. Kappa measure of agreement was used to assess inter-rater reliability at both time points for each question, and ranged from 0.60 to 0.84.

## Results

### Did you enjoy completing the questionnaire?

#### Quantitative results

Of the sample (n = 1973), 1462 (74.10 %) enjoyed the questionnaire at baseline (1430 at follow-up; 72.48 %). Participants who did not enjoy the questionnaire at baseline were more likely to be upset at follow-up, and males were more likely to report that they did not enjoy the questionnaire than females (Table 1). Differences across the four groups were observed on optimism, *F*(3, 1632) = 8.10, *p* < 0.001, η^2^ = 0.02, self-efficacy, *F*(3, 1598) = 6.15, *p* < 0.001, η^2^ = 0.01, problem solving *F*(3, 1649) = 4.86, *p* < 0.002, η^2^ = 0.01, and reference to others *F*(3, 1675) = 5.50, *p* < 0.001, η^2^ = 0.01. In all cases those who enjoyed the questionnaire at both time points reported healthier functioning (e.g. greater self-efficacy) than those who did not enjoy the questionnaire at either time point. Participants who enjoyed the questionnaire at both time points were more optimistic than those who did not enjoy it at follow-up, *F*(1, 1632) = 10.80, *p* = 0.006, and had higher self-efficacy than those who did not enjoy it at baseline, *F*(1, 1598) = 10.71, *p* = 0.006 (Table 2). There was no significant interaction between enjoying the questionnaire and time on any of the dependent variables.

**Table 1:** Differences in demographic factors and suicidal behaviour

	Baseline					Follow up				
	χ^2^	df	N	*p*	V	χ^2^	df	N	*p*	V
Enjoy										
Worry/upset	0.45	1	1829	0.50	0.02	11.31	1	1869	*0.00*	0.08
Gender	7.92	1	1835	0.01	0.07	0.39	1	1881	0.54	0.01
Psych. diagnosis	7.01	1	1816	0.01	0.06	0.74	1	1872	0.39	0.02
Thought of NSSI	0.08	1	1827	0.78	0.01	0.05	1	1872	0.83	0.01
NSSI	3.76	1	1795	0.05	0.05	2.74	1	1875	0.10	0.04
Thought about suicide	5.78	1	1781	0.02	0.06	0.13	1	1860	0.72	0.01
Attempted suicide	1.92	1	1765	0.17	0.03	0.41	1	1858	0.52	0.02
Worry/upset										
Enjoy	0.45	1	1829	0.50	0.02	11.31	1	1869	0.00	0.08
Gender	42.33	1	1906	0.00	0.15	26.54	1	1929	0.00	0.14
Psych. diagnosis	22.29	1	1885	0.00	0.11	36.92	1	1920	0.00	0.14
Thoughts of NSSI	100.65	1	1895	0.00	0.23	101.44	1	1919	0.00	0.23
NSSI	129.25	1	1860	0.00	0.26	119.49	1	1922	0.00	0.25
Thought about suicide	72.37	1	1848	0.00	0.20	81.05	1	1907	0.00	0.21
Attempted suicide	42.64	1	1832	0.00	0.15	23.11	1	1903	0.00	0.11

NB: Girls were more likely to enjoy participation and boys more likely to report being worried or upset. Participants reporting poorer psychosocial functioning (e.g. more distress, suicidal thoughts and behaviour) were more likely to find the questionnaire worrying or upsetting

**Table 2:** Multivariate analysis of variance demonstrates differential relationships between enjoyment and psychosocial functioning over time

Group	Variable	Baseline M (SD)	Follow up M (SD)	*F*	*p*	η^2^
Enjoy baseline and follow up *n* = 1189	Life events	27.68 (5.03)	28.63 (5.41)	50.66	0.00	0.048
	Self-esteem	30.69 (4.93)	30.25 (5.12)	12.21	0.00	0.011
	Self-efficacy	30.29 (3.89)	30.66 (4.02)	10.01	0.00	0.009
	Non-productive coping	48.88 (11.77)	50.05 (11.48)	14.01	0.00	0.013
	Reference to others	50.66 (13.34)	50.93 (13.24)	0.49	0.49	0.000
	Problem solving	66.84 (12.20)	66.24 (11.59)	3.57	0.06	0.003
	Optimism	20.77 (4.27)	20.69 (4.53)	0.51	0.48	0.000
	Alcohol use	1.91 (2.00)	2.66 (2.72)	147.46	0.00	0.113
	Psych. distress	22.59 (6.04)	23.05 (6.26)	5.86	0.02	0.005
	NSSI	0.84 (3.06)	1.12 (3.45)	8.96	0.00	0.008
Enjoy baseline, not follow up *n* = 223	Life events	28.17 (5.60)	28.97 (5.64)	6.61	0.01	0.033
	Self-esteem	30.56 (4.86)	29.68 (5.21)	8.92	0.00	0.041
	Self-efficacy	30.02 (4.02)	29.90 (3.94)	0.25	0.62	0.001
	Non-productive coping	48.78 (11.55)	50.97 (11.42)	6.71	0.01	0.034
	Reference to others	50.68 (12.61)	50.14 (13.10)	0.34	0.56	0.002
	Problem solving	66.27 (10.78)	63.57 (11.50)	12.31	0.00	0.058
	Optimism	20.17 (4.29)	19.30 (4.70)	9.55	0.00	0.044
	Alcohol use	1.83 (2.07)	2.82 (2.90)	41.33	0.00	0.161
	Psych. distress	22.80 (6.18)	23.71 (6.38)	2.82	0.09	0.013
	NSSI	0.74 (2.94)	1.32 (3.74)	7.10	0.00	0.033
Not enjoy baseline, enjoy follow up *n* = 165	Life events	28.53 (6.09)	28.83 (6.04)	0.478	0.49	0.004
	Self-esteem	30.09 (5.40)	30.00 (5.28)	0.06	0.80	0.000
	Self-efficacy	29.14 (4.13)	29.79 (4.14)	3.75	0.06	0.024
	Non-productive coping	49.47 (12.26)	50.56 (13.28)	1.29	0.26	0.009
	Reference to others	48.87 (14.00)	49.25 (14.27)	0.12	0.73	0.001
	Problem solving	64.82 (12.25)	64.82 (12.92)	0.00	1.00	0.000
	Optimism	19.83 (4.04)	19.93 (4.46)	0.11	0.74	0.001
	Alcohol use	2.26 (2.53)	3.16 (3.06)	16.70	0.00	0.096
	Psych. distress	23.67 (7.34)	23.16 (6.43)	0.91	0.30	0.006
	NSSI	1.47 (3.96)	1.67 (4.11)	0.62	0.43	0.004
Not enjoy baseline or follow up *n* = 189	Life events	28.66 (5.56)	29.85 (5.95)	10.12	0.00	0.063
	Self-esteem	30.03 (4.70)	29.26 (5.30)	4.80	0.03	0.029
	Self-efficacy	29.37 (4.70)	29.83 (4.20)	2.01	0.14	0.013
	Non-productive coping	49.55 (11.46)	50.85 (12.26)	2.11	0.15	0.013
	Reference to others	46.94 (14.20)	47.44 (13.64)	0.26	0.61	0.001
	Problem solving	64.47 (14.17)	62.95 (13.13)	2.56	0.11	0.014
	Optimism	19.71 (4.29)	19.33 (4.22)	1.77	0.19	0.010
	Alcohol use	2.14 (2.24)	3.22 (3.13)	37.92	0.00	0.175
	Psych. distress	22.23 (5.62)	23.68 (6.29)	8.55	0.00	0.047
	NSSI	0.98 (3.31)	1.58 (4.02)	5.46	0.02	0.029

When exploring specific negative life events, at baseline, those who reported being in trouble with the police, or physically abused within the last 12 months were less likely to report enjoying the questionnaire (Table 3). No specific life events were associated with enjoying the questionnaire at follow-up. Enjoying the questionnaire at baseline, χ^2^(3) = 6.45, *p* = 0.09, or at follow-up, χ^2^(3) = 4.44, *p* = 0.22, was not related to onset, maintenance or cessation of NSSI over time.

**Table 3:** Differences in specific items on the Life Events Scale at baseline and follow-up

Life event	Baseline				Follow-up			
	Enjoy n (%)		Worry/upset n (%)		Enjoy n (%)		Worry/upset n (%)	
	Yes^a^	No	Yes	No	Yes	No	Yes	No
	n = 1462	n = 373	n = 304	n = 1602	n = 1430	n = 451	n = 235	n = 1694
Problems with schoolwork	χ^2^ = 1.01, *p* = 0.06		χ^2^ **=** *24.66*, *p* = 0.00		χ^2^ = 1.94, *p* = 0.38		χ^2^ **=** *14.94*, *p* = 0.00	
	624 (42.68)	167 (44.77)	170 (55.92)	647 (40.38)	719 (50.28)	242 (53.66)	147 (62.55)	840 (49.59)
Making/keeping friends	χ^2^ = 1.03, *p* = 0.06		χ^2^ = *31.78*, *p* = 0.00		χ^2^ = 0.66, *p* = 0.72		χ^2^ = *23.75*, *p* = 0.00	
	293 (20.04)	83 (22.25)	89 (29.28)	298 (18.60)	307 (21.47)	102 (22.62)	79 (33.62)	337 (19.89)
Arguments/fights with friends	χ^2^ = 3.40, *p* = 0.02		χ^2^ = *22.05*, *p* = 0.00		χ^2^ = 3.29, *p* = 0.19		χ^2^ = *17.61*, *p* = 0.00	
	370 (25.31)	108 (28.95)	106 (34.87)	383 (23.91)	372 (26.01)	137 (30.38)	90 (38.30)	429 (25.32)
Problems with girl/boyfriend	χ^2^ = 1.46, *p* = 0.48		χ^2^ = *32.73*, *p* = 0.00		χ^2^ = 0.55, *p* = 0.76		χ^2^ = *23.57*, *p* = 0.00	
	126 (8.62)	38 (10.19)	53 (17.43)	117 (7.30)	172 (12.03)	60 (13.30)	49 (20.85)	189 (11.16)
Bullied at school	χ^2^ = 1.45, *p* = 0.48		χ^2^ = *14.40*, *p* = 0.00		χ^2^ = 0.03, *p* = 0.98		χ^2^ = *31.92*, *p* = 0.00	
	216 (14.77)	59 (15.82)	65 (21.38)	223 (13.92)	181 (12.66)	58 (12.86)	57 (24.26)	193 (11.39)
Parents separated or divorced	χ^2^ = 1.15, *p* = 0.56		χ^2^ = 6.82, *p* = 0.03		χ^2^ = 0.35, *p* = 0.84		χ^2^ = *8.53*, *p* = 0.01	
	42 (2.87)	11 (2.95)	15 (4.93)	41 (2.56)	41 (2.87)	12 (2.66)	12 (5.11)	42 (2.48)
Serious arguments with parents	χ^2^ = 6.71, *p* = 0.04		χ^2^ = *55.76*, *p* = 0.00		χ^2^ = 11.43, *p* = 0.03		χ^2^ = *23.26*, *p* = 0.00	
	375 (25.65)	119 (31.90)	133 (43.75)	379 (23.66)	443 (30.98)	149 (33.04)	107 (45.53)	504 (29.75)
Parents fights	χ^2^ = 2.92, *p* = 0.40		χ^2^ = *49.66*, *p* = 0.00		χ^2^ = *4.96*, *p* = 0.08		χ^2^ = *22.92*, *p* = 0.00	
	251 (17.17)	75 (20.11)	92 (30.26)	246 (15.36)	277 (15.87)	94 (20.84)	72 (30.64)	314 (18.54)
You or family member: serious illness or accident	χ^2^ = 2.28, *p* = 0.32		χ^2^ = *13.14*, *p* = 0.00		χ^2^ = 1.40, *p* = 0.50		χ^2^ = *30.45*, *p* = 0.00	
	322 (22.02)	91 (24.40)	90 (29.61)	339 (21.16)	377 (26.36)	131 (29.05)	98 (41.70)	425 (25.09)
Close friends: serious illness or accident	χ^2^ = 2.28, *p* = 0.32		χ^2^ = *14.59*, *p* = 0.00		χ^2^ = 6.44, *p* = 0.04		χ^2^ = *30.00*, *p* = 0.00	
	170 (11.63)	53 (14.21)	55 (18.09)	172 (10.74)	196 (13.71)	77 (17.07)	61 (25.96)	217 (12.91)
Death in immediate family	χ^2^ = 1.80, *p* = 0.41		χ^2^ = 4.90, *p* = 0.08		χ^2^ = 1.92, *p* = 0.37		χ^2^ = 6.80, *p* = 0.03	
	35 (2.40)	13 (3.49)	13 (4.28)	37 (2.31)	28 (1.96)	12 (2.66)	10 (4.26)	30 (1.77)
Death of someone close	χ^2^ = 3.47, *p* = 0.18		χ^2^ = *14.93*, *p* = 0.00		χ^2^ = 2.21, *p* = 0.33		χ^2^ = *19.61*, *p* = 0.00	
	235 (16.07)	69 (18.50)	72 (23.68)	240 (14.98)	242 (16.92)	86 (19.07)	64 (27.23)	267 (15.76)
Family or friends suicide	χ^2^ = 4.54, *p* = 0.10		χ^2^ = *22.08*, *p* = 0.00		χ^2^ = 1.16, *p* = 0.56		χ^2^ = *20.16*, *p* = 0.00	
	25 (1.71)	12 (3.22)	12 (3.95)	24 (1.50)	54 (3.78)	19 (4.21)	19 (8.09)	53 (3.13)
Family attempt suicide or self-harm	χ^2^ = 4.16, *p* = 0.13		χ^2^ = *86.62*, *p* = 0.00		χ^2^ = 1.89, *p* = 0.39		χ^2^ = *54.23*, *p* = 0.00	
	44 (3.01)	6 (1.61)	27 (8.88)	25 (1.56)	54 (3.78)	18 (3.99)	24 (10.21)	49 (2.89)
Friend attempt suicide or self-harm	χ^2^ = 2.21, *p* = 0.33		χ^2^ = *99.44*, *p* = 0.00		χ^2^ = 1.51, *p* = 0.47		χ^2^ = *80.69*, *p* = 0.00	
	220 (15.05)	56 (15.01)	101 (33.22)	186 (11.61)	288 (20.14)	93 (20.62)	93 (39.57)	298 (17.59)
Physically abused	χ^2^ = *9.50*, *p* = 0.00		χ^2^ = *14.51*, *p* = 0.00		χ^2^ = 1.48, *p* = 0.48		χ^2^ = *17.61*, *p* = 0.00	
	16 (1.09)	8 (2.14)	9 (2.96)	14 (0.87)	21 (1.47)	6 (1.39)	8 (3.40)	20 (1.18)
Trouble with police	χ^2^ = *12.89*, *p* = 0.00		χ^2^ = *17.72*, *p* = 0.00		χ^2^ = 2.01, *p* = 0.37		χ^2^ = 1.89, *p* = 0.39	
	15 (1.03)	12 (3.22)	9 (2.96)	20 (1.25)	26 (1.81)	12 (2.66)	6 (2.55)	33 (1.95)
Worries about sexual orientation	χ^2^ = 3.37, *p* = 0.19		χ^2^ = *19.13*, *p* = 0.00		χ^2^ = 0.77, *p* = 0.68		χ^2^ = *53.67*, *p* = 0.00	
	88 (6.02)	18 (4.83)	32 (10.53)	75 (4.68)	29 (2.03)	32 (7.09)	43 (18.30)	104 (6.14)
Forced to engage in sexual activity	χ^2^ = 1.39, *p* = 0.50		χ^2^ = *20.05*, *p* = 0.00		χ^2^ = 1.23, *p* = 0.54		χ^2^ = *54.70*, *p* = 0.00	
	19 (1.30)	6 (1.61)	11 (3.62)	14 (0.87)	29 (2.03)	10 (2.22)	14 (5.96)	25 (1.48)
Other distressing event	χ^2^ = 0.41, *p* = 0.81		χ^2^ = *87.57*, *p* = 0.00		χ^2^ = 0.33, *p* = 0.85		χ^2^ = *108.57*, *p* = 0.00	
	199 (13.61)	53 (14.21)	90 (29.61)	170 (10.61)	247 (17.27)	75 (16.63)	93 (39.57)	237 (13.99)

NB: At both time points, being worried or upset is associated with experiencing more negative life events

^a^Percentages represent the proportion of participants responding yes or no to this question, who reported experiencing the given life event in the previous 12 months

#### Thematic analysis

Five themes were evident among those who enjoyed the questionnaire; (1) understanding and reflection, (2) help is available/helping others, (3) that it was fun or enjoyable, (4) self-expression, (5) getting out of class. Two major themes emerged among those that did not enjoy the questionnaire; (1) boredom/inconvenience, or (2) negative experiences (Table 4).

**Table 4:** Exemplars reflecting key themes extracted in qualitative analysis

Theme	Exemplars
Enjoy the questionnaire	
Understanding and reflection	It allowed me to reflect on my positive thoughts and how I view myself as an individual Knowing I haven’t tried any illegal substances or at least reading I haven’t made me feel good On the last page my head started running over all the pleasant memories I was writing about It gave me a better understanding of myself It helped me to understand more about the mental health problems of young people and helped me understand more about me
Help is available/helping others	I hope that what I have answered can contribute even just a little to the research I know it will help research into emotional problems with teenagers providing solutions to overcome their issues Made me feel that there is help out there, we just need to talk about it I did enjoy this because it makes me know that someone is trying to help
Fun and enjoyable	It was interesting and I enjoy filling out questionnaires Because I like being asked questions about me Yes, it was enjoyable. More fun than what I thought It was a change and I enjoyed it
Expressing themselves	Because I can release my feelings, emotions and thoughts and know they will stay anonymous I can tell the truth and not get into trouble I knew I could be completely honest and it felt good to express some things that I had not in any other way
Getting out of class	I got out of geography class and got a show bag It meant we don’t have to do work Because I won’t have to do schoolwork and listen to the teacher…
Not enjoy the questionnaire	
Boredom/inconvenience	I didn’t have a problem with my life so it wasn’t really effective for me Didn’t really relate to any of my problems I sometimes found it hard to understand what the question was asking for example, I have never thought about committing suicide so I found it strange answering questions about that topic Cause it was boring… It took ages. I was meant to be in the library doing assignments and studying Missed studying for science test coming up
Negative experience	Shows the bad side of me It made me think about my problems too much I did not realise other people had some of the worries mentioned It made me sad for others
Worried or upset	
Personal experience/feelings	It was just upset remembering things that have happened I realised that maybe I do need help I feel like there might be something wrong with me Recently someone in my community committed suicide so the questions relating to that were upsetting The self-harm/suicide part and the bullying part and the sexuality part
Relationships with others	Brought back memories of awkwardness with my ex-girlfriend I don’t have a relationship with my dad, which makes me sad I didn’t like the questions about family because my relationship with my family is great When I shared thoughts about my friends, I wished hard that I had better friends …it’s hard because I have a really close friend who does it [self-injures] and I can’t stop him
Awareness of others’ issues	Some people my age actually have these problems That there are a lot of mental problems out there The thought that young people take their lives The drinking and drugs, it just worried me that people do that to themselves
Confidentiality	I was worried this questionnaire wouldn’t be confidential … I might be identified by my answers … you might contact the school about me I… am worried about my parents finding out

### Positive responses

1. *Understanding and reflection* At both baseline and follow-up the most common reason given for enjoying the questionnaire was that participants experienced a greater understanding of themselves and others (baseline n = 511; follow-up n = 488; ~13 % of total sample). These participants stated that they enjoyed the questionnaire because they had given positive responses and felt good about themselves. Others found that the questionnaire helped them to experience good memories, and reminded them of good things in life.
2. *Help is available/helping others* 12.8 % (n = 234) at baseline and 15.5 % (n = 286) at follow-up reported they felt they were helping others, specifically the researchers and other adolescents in need. In addition, some responded that through the questionnaire, they now felt that help was available to them.
3. *Enjoyable and interesting* At both baseline (n = 268, 14.6 %) and follow-up (n = 237, 12.8 %) several participants enjoyed the questionnaire simply because it was fun or interesting. Many stated that “I found it interesting”. Several reported enjoying the questionnaire because they like questionnaires.
4. *Self*-*expression* At baseline 176 (9.6 %) and at follow-up 126 (6.8 %) indicated that the questionnaire gave them the opportunity to express their feelings without fear of people finding out about their behaviour, or judging them for it.
5. *Getting out of class* Only 5.2 % (n = 96) at baseline and 7.7 % (n = 143) at follow-up reported enjoying the questionnaire because they got out of class.

### Negative responses

1. *Inconvenience/irrelevance* At both baseline and follow-up, the most common reason for not enjoying the questionnaire was that students found it inconvenient or irrelevant to them (baseline n = 255, 13.9 %; follow-up n = 300, 16.2 %). Some felt that they didn’t have anything to talk about, where others clearly felt that whilst they had problems, the questionnaire didn’t address them. Finally, others commented that the questionnaire was too long, or that they didn’t enjoy it because they would have preferred to be doing something else.
2. *Negative experiences* Only 5.4 % (n = 98) at baseline and 5 % (n = 93) at follow-up reported a negative experience associated with participation. Some indicated answering questions made them upset about their own problems and concerns, whereas others gained an understanding of what others go through and this made them feel uncomfortable or unhappy.

### Did anything in this questionnaire worry or upset you?

#### Quantitative results

At baseline, 304 participants (15.40 % of total sample) found the questionnaire worrying or upsetting which decreased to 235 (12.18 %) participants at follow-up. Males were less likely to be upset by the questionnaire than females. At both baseline and follow-up participants were more likely to report feeling worried or upset if they had a previous diagnosis of emotional or behavioural problems. Those who were worried or upset were also more likely to have thought about harming themselves, to have told someone about it, to have self-injured, thought about and attempted suicide at both baseline and follow-up (Table 1).

When considering changes in the reaction to the questionnaire over time, between group effects were evident for all variables except reference to others (all *p* < 0.01). People who were upset at baseline but not follow-up had poorer functioning on all variables except alcohol use and relying on others to cope, (all *p* < 0.001), than those who were not upset at either time point. Participants upset at baseline only also reported more psychological distress, less self-esteem, more life events, and higher self-injury scores (all *p* < 0.001) than those who were upset at both time points. Participants upset at both time points reported less problem solving, more drinking, and higher self-injury scores, than those not upset at either time (all *p* < 0.001). Participants upset at follow-up but not baseline exhibited less problem solving, *F*(1, 1742) = 18.87, *p* < 0.001, than participants who were not upset by the questionnaire at either time (Table 5).

**Table 5:** Multivariate analysis of variance demonstrates differential relationships between being worried or upset and psychosocial functioning over time

Group	Variable	Baseline M (SD)	Follow up M (SD)	*F*	*p*	η^2^
Upset baseline and follow up *n* = 96	Life events	33.23 (6.96)	34.38 (6.65)	4.15	0.05	0.050
	Self-esteem	26.68 (5.75)	25.73 (6.02)	3.55	0.06	0.038
	Self-efficacy	27.40 (4.44)	28.84 (4.50)	11.36	0.00	0.115
	Non-productive coping	56.23 (12.34)	58.79 (11.15)	50.4	0.03	0.056
	Reference to others	49.39 (12.69)	51.67 (13.22)	2.42	0.12	0.026
	Problem solving	60.40 (13.12)	60.00 (13.76)	0.09	0.77	0.001
	Optimism	17.66 (4.69)	17.66 (5.63)	0.000	1.00	0.000
	Alcohol use	2.30 (2.67)	3.72 (3.33)	15.97	0.00	0.154
	Psych. distress	29.14 (8.15)	29.44 (7.92)	0.11	0.75	0.001
	NSSI	4.14 (5.90)	5.58 (6.00)	6.75	0.01	0.074
Upset baseline, not follow up *n* = 197	Life events	30.51 (5.61)	30.70 (5.73)	0.28	0.60	0.002
	Self-esteem	28.50 (5.15)	28.87 (4.89)	1.63	0.20	0.009
	Self-efficacy	28.94 (4.24)	29.78 (4.03)	7.29	0.01	0.040
	Non-productive coping	53.79 (12.28)	53.18 (10.79)	0.57	0.45	0.003
	Reference to others	50.24 (13.58)	51.16 (13.62)	1.10	0.30	0.006
	Problem solving	63.40 (11.78)	63.39 (11.54)	0.000	0.98	0.000
	Optimism	18.82 (4.41)	18.86 (4.58)	0.02	0.90	0.000
	Alcohol use	2.26 (2.23)	3.18 (2.89)	30.15	0.00	0.136
	Psych. distress	25.64 (7.00)	24.24 (6.16)	7.02	0.01	0.036
	NSSI	1.81 (4.35)	2.00 (4.35)	0.47	0.49	0.003
Not upset baseline, upset follow up *n* = 131	Life events	28.81 (4.97)	31.85 (5.75)	37.67	0.00	0.260
	Self-esteem	29.82 (4.81)	27.33 (5.44)	32.37	0.00	0.217
	Self-efficacy	30.04 (4.19)	29.19 (3.88)	4.92	0.03	0.042
	Non-productive coping	50.17 (10.87)	54.51 (11.88)	15.67	0.00	0.127
	Reference to others	50.83 (13.65)	50.79 (12.19)	0.001	0.98	0.000
	Problem solving	64.35 (12.34)	60.66 (12.07)	11.90	0.00	0.086
	Optimism	20.34 (4.07)	18.22 (4.39)	31.18	0.00	0.206
	Alcohol use	1.90 (2.10)	3.02 (3.01)	25.30	0.00	0.167
	Psych. distress	23.86 (5.86)	27.58 (7.42)	28.51	0.00	0.192
	NSSI	0.72 (2.93)	2.54 (4.86)	19.87	0.00	0.140
Not upset baseline or follow up *n* = 1446	Life events	27.09 (4.79)	27.88 (5.03)	42.07	0.00	0.034
	Self-esteem	31.14 (4.67)	30.69 (4.88)	15.80	0.00	0.012
	Self-efficacy	30.37 (3.86)	30.66 (3.97)	8.12	0.00	0.006
	Non-productive coping	47.61 (11.37)	48.85 (11.39)	16.98	0.00	0.013
	Reference to others	49.87 (13.38)	49.87 (13.44)	0.00	0.99	0.000
	Problem solving	67.28 (12.06)	66.30 (11.76)	10.48	0.00	0.008
	Optimism	20.90 (4.12)	20.81 (4.37)	0.84	0.36	0.001
	Alcohol use	1.86 (2.00)	2.65 (2.76)	189.53	0.00	0.119
	Psych. distress	21.74 (5.44)	22.32 (5.70)	12.03	0.00	0.009
	NSSI	0.57 (2.53)	0.76 (2.95)	8.16	0.00	0.006

Changes in the variables over time varied according to whether participants were upset by the questionnaire for optimism, psychological distress, self-esteem, self-efficacy, life events, non-productive coping, and self-injury (Table 5; all *p* < 0.001). Of note, those who were not upset at baseline but were upset by the questionnaire at follow-up demonstrated deterioration in functioning (e.g. less adaptive coping, more psychological distress) over time. Conversely those who were upset by the questionnaire at baseline but not at follow-up demonstrated increased self-efficacy and decreased psychological distress over time.

With regard to specific life events, at both time points, those who reported feeling worried or upset were more likely to report having experienced a range of events, primarily to do with close relationships (e.g. problems with friends and parents) as well as suicidal behaviour among friends and family (Table 3). Finding the questionnaire upsetting at baseline was also related to the course of NSSI over the study period, χ^2^(3) = 155.37, *p* < 0.001. Specifically, participants reporting maintenance (standardised residual = 8.4) or cessation of NSSI (standardised residual = 6.4) were more likely to find the questionnaire upsetting at baseline. Of those who were upset at baseline, 8 % later engaged in NSSI, but this was not a significant relationship (1.3 % of the total sample reported being upset at baseline and onset of NSSI over the study period; 3.4 % who were not upset at baseline reported onset of NSSI). Similar results were observed for participants who were upset by the questionnaire at follow-up, χ^2^(3) = 152.51, *p* < 0.001. Specifically, adolescents who maintained their NSSI over time (standardised residual = 6.5), and those who first commenced self-injury between baseline and follow-up (standardised residual = 8.0) were more likely to be upset by the questionnaire at follow-up, while cessation of NSSI was not associated with being upset by the questionnaire.

#### Thematic analysis

When asked why they felt worried or upset, four themes emerged; (1) personal experience/feelings, (2) relationships with others, (3) awareness of other people’s issues, (4) confidentiality.

1. *Personal experience/feelings* At both time points (baseline n = 80; follow-up n = 85; 4 % of total sample), the most common reason for reporting feeling upset or worried was that the questionnaire prompted memories they would prefer to avoid. Most frequently, these life events were related to death or suicide. Many felt that the questions asked were too personal, but others felt concerned that they themselves might need help. Finally, several participants expressed a dislike for themselves, and were worried that they might be judged based on their responses.
2. *Relationships with others* Participants reported feeling worried or upset in relation to their interpersonal relationships (baseline n = 72; follow-up n = 59; 3 % of total sample), including relationships with friends, family, and romantic partners. Some expressed concerns about their friends’ behaviour, or felt that the friends they had were not very good friends. Romantic relationships, while less frequently identified than family or friend relationships, caused some participants concern.
3. *Awareness of others’ issues* A small number of participants reported increased awareness of the problems or emotional concerns that others might have (baseline n = 42; follow-up n = 23; 1.6 % of total sample). Most of these responses indicated a new awareness that some young people engage in harmful behaviour, including drinking, taking drugs and NSSI, and that some consider suicide.
4. *Confidentiality* A relatively small proportion of participants expressed concerns that others may find out about their responses, 17 at baseline and 9 at follow-up (0.6 % of total sample).

## Discussion

Research exploring risk and protective factors for the development of later mental health problems, among currently healthy adolescents, is imperative to the development of evidence-based prevention and early intervention programs for mental illness. However ethical concerns arise regarding the potential for sensitive questions to evoke distress, particularly in young samples. We explored whether participation in sensitive research is perceived as enjoyable or upsetting to young people, and the reasons for these reactions. We also examined whether changes in psychosocial functioning over time were related to changes in perceptions of research participation. Finally we assessed whether enjoying the questionnaire or finding it upsetting was associated with the course of NSSI over time (i.e. onset, maintenance or cessation of NSSI).

More participants reported positive outcomes than negative, and these were largely related to experiencing a greater understanding of self and others, or feeling altruistic about helping other people. Where participants reported negative outcomes, these were related to disinterest, although some students also reported being upset by the content of questions related to recent life events. Changes in response to the questionnaire were accompanied by predictable changes in psychosocial functioning; greater enjoyment was accompanied by improved psychosocial functioning while negative reactions were accompanied by deteriorating functioning. We failed to ask young people whether they regretted participation, but fewer than 1 % of participants (or their parents) failed to consent to participation at follow-up suggesting that even those who were upset at baseline were willing to complete the questionnaire a second time [11, 12].

That fewer participants reported being worried or upset by the questionnaire at follow-up may indicate that the adaptive coping and emotion regulation skills typically developed throughout adolescence protect older students from adverse emotional reactions. If so, additional care should be taken to minimise potential distress and address duty of care with younger adolescents. Conversely, prior exposure to the questions may have minimised the emotional impact of the items.

### Implications

Our findings suggest that young people *do* enjoy participating in psychological research. School staff may take comfort in knowing that not only are their students contributing to scientific knowledge, but that they also gain insight about their own behaviour, and that of their peers. In our study students became more aware of their own emotional struggles, and some indicated that although they did not know where to seek support for emotional difficulties prior to the study, through participation (and arguably our provision of mental health materials) they now knew that help was available, and where to seek it. Conducting research with adolescents could promote opportunities for help-seeking, delivering intervention earlier than might otherwise have occurred, and improving the prognosis for young people who are distressed.

Our results also suggest that young people can, and do, become upset when asked to respond to questions about their mental health, particularly if the questions touch on recent negative life events. In addition to minimising the potential for such distress to occur, resources need to be made available to young people who experience distress as a result of questionnaire completion. The fact that some participants expressed concerns regarding confidentiality, despite our provision of both written and verbal assurance, suggests researchers need to take additional steps to ensure that young people clearly understand how the researchers will utilise their data. Arguably, if participants were not clear regarding confidentiality, they may also have been unclear regarding other aspects of the study. This has important implications for the ability to provide informed consent and stresses the responsibility of researchers to ensure all participants are fully informed about the nature of the research.

### Limitations and future research

The relatively low response rate, while consistent with similar Australian research requiring both active parental and active child consent [15], is lower than other longitudinal research and suggests bias in the sample. Of note, given the sensitive nature of many of the questions we asked, the lack of representativeness of the sample may mask important relationships between NSSI, psychological distress and reflections on survey participation. Comments from parents and teachers suggest that parents who were concerned about their child’s mental health were reluctant to consent to their child’s participation. That the vast majority of the sample enjoyed the questionnaire and were not upset by it may be an artefact of this selection bias.

While one of the key concerns in conducting research with youth is the potential for iatrogenic effects we are not able to directly assess this. Alcohol use and NSSI did increase over time, for all groups, but we cannot determine whether this is a consequence of being exposed to the questionnaire items or whether it is a natural artefact of progression through adolescence. Our analyses suggest that people who are upset by research participation are more likely to self-injure, and that a percentage do later engage in NSSI. However it is not possible with the current design to establish a causal relationship. Onset of NSSI over the study period is likely an accumulation of adverse life events, poor psychosocial functioning and perhaps reflection on this through answering these questions. Future research is needed to explicitly determine whether responding to questionnaire items regarding high risk behaviour confers risk of NSSI and other adverse effects among participants, under what conditions iatrogenic effects might be observed, and how to minimise these effects.

Although the majority of our measures demonstrated reliability, the ‘reference to others’ subscale of the Adolescent Coping Scale did not, which may account for the failure to find significant effects. Similarly, the global assessment of distress or enjoyment of the questionnaire precludes examination of whether students might respond differentially to different parts of the questionnaire. Further work regarding which type of questions might have most potential to cause distress, and whether this overrides any benefits of participation, is warranted. Finally, our question regarding distress combined the terms worry and upset, arguably two different emotional reactions. Disentangling how participants experience distress would be important for better assessing the risk of psychological harm as a result of research participation.

## Conclusions

In exploring the reactions of adolescents to responding to sensitive research questions, we have confirmed previous findings that research can have both benefits and risks for participants. Encouragingly, the majority of school-based adolescents enjoy research participation and cite altruistic reasons for participation. However, a minority of youth, particularly those who are already experiencing distress, are temporarily distressed by responding to questions about mental health. Researchers are encouraged to emphasise the benefits of research participation, while also continuing to work to minimise the potential for distress and implement appropriate duty of care protocols.
